# The Biology and Thermal Requirements of the Fennel Aphid *Hyadaphis foeniculi* (Passerini) (Hemiptera: Aphididae)

**DOI:** 10.1371/journal.pone.0100983

**Published:** 2014-07-08

**Authors:** José B. Malaquias, Francisco S. Ramalho, Aline C. S. Lira, Flávia Q. Oliveira, Francisco S. Fernandes, Wesley A. C. Godoy, José C. Zanuncio

**Affiliations:** 1 Unidade de Controle Biológico, Embrapa Algodão, Av. Osvaldo Cruz, Campina Grande-PB, CEP, Brazil; 2 Universidade Estadual Paulista –UNESP, Jaboticabal, SP, Brazil; 3 Escola Superior de Agricultura Luiz de Queiroz – USP, Piracicaba, SP, Brazil; 4 Univiversidade Federal de Viçosa, Viçosa, MG, Brazil; CNRS, France

## Abstract

The relationship between the insect development rate and temperature was established very early and represents an important ecological variable for modeling the population dynamics of insects. The accurate determination of thermal constant values and the lower and upper developmental thresholds of *Hyadaphis foeniculi* (Passerini) (Hemiptera: Aphididae) on fennel (*Foeniculum vulgare* Miller (Apiales: Apiaceae)) crops would obviously benefit the effective application of control measures. This paper is a study of the biology and thermal requirements of *H. foeniculi*. Winged insects were collected from fennel crops at the Embrapa Algodão in Campina Grande, Paraíba. Nymphs (age ≤24 h) produced by winged insects were subjected to constant temperatures of 15, 20, 25, 28, 30 or 33°C, a photophase of 12 h and a relative humidity of 70±10%. The results of the study showed that at temperatures between 15 and 30°C, *H. foeniculi* nymphs were able to develop normally. The four instars were found at all temperatures tested. However, temperatures of 3 and 33°C were lethal to the nymphs. The nymph stage development time varied from 5 (30°C) to 19 (15°C) days. The influence of temperature on the development time is dependent on the instar. The base temperature (*Tb*) and the thermal constant (*K*) for the nymph stage were estimated at 11.2°C and 107.5 degree-days, respectively. The shortest nymph development stage was observed at 30°C, and the highest nymph viability (85.0%) was observed at 28°C. This information can be used for developing phenological models based on the temperature and development rate relationships so that outbreaks of *H. foeniculi* in the fennel crop can be predicted, therefore improving the application of control programs targeting this fennel pest.

## Introduction

Fennel (*Foeniculum vulgare* Miller (Apiales: Apiaceae)) is native to coastal Mediterranean regions [1]–[3] and occurs naturally throughout Europe and North America. It was introduced into Brazil by the first settlers and rapidly spread through the states of Bahia, Sergipe, Paraíba and Pernambuco [4]. Fennel is known to have therapeutic properties (digestive, diuretic and anti-inflammatory) [5] as well as culinary uses (soups, pastries and cakes). It also has insecticidal [6]–[8] and fungicidal [8] activity. Therefore, fennel has a guaranteed market in northeastern Brazil and is important for family farming in the region [9].

Factors that impair fennel production and seed quality in Brazil include insect pests, especially the aphid *Hyadaphis foeniculi* (Passerini) (Hemiptera: Aphididae). *H. foeniculi* is a cosmopolitan species and a vector for at least 12 types of viruses, including mosaic potyvirus, yellow luteovirus and carlavirus [4]. Because it continually sucks sap, it causes flowers and fruits to wilt and dry out [7]. It also produces a secretion known as “honeydew”, which is favorable to the development of *Capnodium* spp. fungus, leading to the formation of sooty mold [10]; this mold prevents the plant from transpiring and reduces the photosynthesis area, weakening the plant [11]. In the state of Paraíba, *H. foeniculi* usually reproduces during hot periods, forming colonies inside flowers [7]. The fluctuation of the aphid population is highly seasonal, and populations can vary from one year to the next. This variation is related to the species and their feeding habits and to the availability and phenology of the host plant [10].

In northeastern Brazil, fennel is intercropped with various other crops, such as colored-fiber cotton (*Gossypium hirsutum* Linné) [12]–[14]. Intercropping fennel with colored-fiber cotton has contributed to a 62% drop in the damage caused by *H. foeniculi* to the fennel crop [9].

*Hyadaphis foeniculi* is considered a major insect pest of fennel [9], mainly attacking the flowers, fruits and leaves. By continually sucking the sap, it causes these organs to wilt and dry out, impairing the fennel seed [4] and damaging up to 80% of the yield [9] in non-intercropped fennel. Aphids are mainly controlled by chemical means. However, this method can prove ineffective if implemented at the wrong time of year. Predicting pest attacks based on the pest's thermal requirements could improve the control efficiency. The use of laboratory degree-day models has helped in pest management programs [15] and has revealed the aphid's population dynamics so that sampling times can be more efficiently determined and the number of pest generations defined.

Climatic factors considerably influence the aphid pest populations. Temperature is considered the most important abiotic factor affecting physiology [16], longevity, development rates, aphid reproduction rates [17] and, consequently aphid population dynamics [18]. The relationship between temperature and insect development has been acknowledged for some time as an important ecological variable in modeling insect pest population dynamics [18], [19]. Precise estimates of insect thermal requirements based on the thermal constant expressed in degree-days (DD) and development rates are important factors in phenological model-based pest management programs [20]. Phenological models based on temperature dependence relationships can be used to accurately investigate geographic distribution, population dynamics and management strategies [21]. They also help in predicting pest outbreaks in the field and determining the best time to control them and enhancing laboratory breeding techniques based on predictions of survival, instar duration and reproductive capability [15]–[16].

Determining the thermal thresholds that affect the biological activities of the insects is an important step in understanding the effects of environmental variations on their fitness and population dynamics [22]. Little is known about the thermal tolerance of *H. foeniculi* or the bioecology of this aphid [13], yet this information could allow us to optimize control strategies. Therefore, the aim of this study was to investigate the biological responses of *H. foeniculi* to different temperatures and determine its thermal requirements under laboratory conditions. These estimates (*Tb* and *K*) may be useful for predicting the seasonal abundance of the *H. foeniculi* under field conditions and, therefore, would contribute to improving the application of control programs targeting this fennel pest.

## Materials and Methods

### Insects and fennel cultivar

Winged fennel aphids (*H. foeniculi*) were collected in crops of fennel (*F. vulgare*) planted with the ‘Montadas’ cultivar at the Embrapa Algodão research facility, Campina Grande, Paraíba, Brazil. The aphids were kept in incubators at 25°C, a relative humidity of 70±10% and a photophase of 12 h. They were placed in 100-ml plastic containers each containing an 8-cm leaf taken from a fennel plant in the vegetative state.

An end of each leaf was kept in a 2.5-ml plastic tube (usually used for dental anesthetic) filled with water to keep the leaf fairly turgid. The end of the plastic tube was sealed with water-absorbent cotton wool to prevent leakage. The tube was inserted into the middle of the plastic container through a circular hole 3.1 cm in diameter. The water and leaves were replaced every day.

### Biology and thermal time bioassays

The nymphs (age ≤24 h) produced by the winged aphids were subjected to constant temperatures of 15, 20, 25, 28, 30 or 33°C, a relative humidity of 70±10% and a 12-h photophase. For each temperature, the experiment began with 200 recently hatched nymphs, split into four replications of 50 nymphs. All experimental units of each treatment were done at the same time and performed in a single chamber. All of the experimental units of each treatment were conducted at the same time in a single chamber. The insects were kept in 100-ml plastic containers, each containing an 8-cm leaf taken from the “Montadas” cultivars of *F. vulgare* in the vegetative state. A piece of black paper was placed inside each container to make the exuvia (remains of the exoskeleton) more visible. The nymphs were given fresh water and leaves every day.

Observations were made at 12-h intervals using a stereoscopic microscope. The duration and survival data were recorded for each instar and nymph phase. The duration of each instar was determined based on ecdysis (molting) and the consequent production of the exuvia. The duration of the nymph phase was quantified from hatching until the emergence of the adult. The adults were identified by the presence of corniculi; furthermore, the production of offspring characterized the adult stage.

### Data analysis and statistics

The nymph development and survival time data were subjected to analysis of variance using the GLM procedure [23], and the means were compared using the Student-Newman-Keuls test (*P* = 0.05). For the purposes of this procedure, the survival data for each instar and the nymph stage were transformed into 1/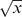 [24], and the nymph development times were transformed into log (x) [24].

Based on the nymph development and survival data, regression curves were generated for each instar using PROC GENMOD [23]. The aphid survival data at each temperature were analyzed by logistic regression, calculating the individual survival probability for each instar during the nymph stage, in line with the binomial probability distribution. The development base temperature (*T_b_*) and the thermal constant (*k* (degree-days)) for each instar and for the nymph stage were estimated by the hyperbole method [25]. To offset the non-linearity effects on transforming the development time into a rate [26], the mean development rates were calculated using the following equation:
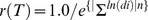
where r(T) =  development rate, di =  individual observations of development time in days and n =  number of observations made.

## Results

Temperatures between 15 and 30°C allowed the development of nymphs of all instars of *H. foeniculi*. Irrespective of temperature, four instars were recorded in this range. Temperature of 3 and 33°C were lethal to the insect. The instar-temperature interaction for nymph survival was significant (*F_12, 57_* = 193.41; *P*<0.0001) (Table 1).

**Table 1:** Summarized models of the effects of temperature^1^ and instar on the development time^2^ and survival^3^ of instars of the fennel aphid *H. foeniculi* using two-way analysis of variance (ANOVA).

Source	Model	DF	F ratio	Pr>F
Development (d)	Model	22	61.31	0.0001
	Temperature (T)	4	296.50	0.0001
	Instar (I)	3	28.23	0.0001
	T×I	12	6.43	0.0001
Survival (%)	Model	22	257.82	0.0001
	Temperature (T)	4	294.04	0.0001
	Instar (I)	3	721.11	0.0001
	T×I	12	193.41	0.0001

1 Temperatures: 15°C, 20°C, 25°C, 28°C, 30°C and 33°C.

2 Development: data transformed into log (x).

3 Survival: data transformed into 1/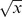.

The survival of *H. foeniculi* ranged from 65.00% (15°C) to 92.45% (25°C) (*F_4, 12_* = 758.23; *P*<0.0001) in the 1st instar; from 80.29% (15°C) to 97.27% (28°C) (*F_4, 12_* = 192.34; *P*<0.0001) in the 2nd instar; from 76.00% (25°C) to 100.00% (28 and 30°C) (*F_4, 12_* = 879.04; *P*<0.0001) in the 3rd instar and from 89.87% (20°C) to 100.00% (25°C) (*F_4, 12_* = 41.22; *P*<0.0001) in the 4th instar (Table 2). The nymph survival was, on average, two-fold higher at the temperature of 28°C compared with the lowest temperature (15°C).

**Table 2:** Survival (%, mean ± SE) of the nymphs in different instars and the nymph stage of *H. foeniculi* at five constant temperatures, a photophase of 12 h and a relative humidity of 70±10%.

Temperature (°C)	Instar^1^				F; Pr	Nymphal stage
	1st	2nd	3rd	4th		(%)^2^^,^ 3
15	65.00±0.40 Dd	80.29±0.55 Dc	90.25±0.30 Bb	94.30±0.71 Ba	= 380.50; <0.0001	44.42±4.42E
20	71.75±0.66 Bc	82.69±0.23 Cb	87.62±0.85 Ca	89.87±0.22 Ca	= 26.82; <0.0001	46.71±4.61D
25	92.45±0.24 Ab	91.97±0.49 Bb	76.09±0.82 Dc	100.00±0.00 Aa	= 1,506.10; <0.0001	64.71±5.75B
28	92.32±0.45 Ad	97.27±0.21 Ab	100.00±0.00 Aa	94.48±0.20 Bc	= 435.86; <0.0001	84.95±7.02A
30	67.62±.62 Cd	91.52±0.89 Bc	100.00±0.00 Aa	95.50±0.02 Bb	= 701.21; <0.0001	59.13±7.02C
F; Pr	= 758.23; <0.0001	= 192.34; <.0001	= 879.04; <0.0001	= 41.22; <0.0001	-	= 379.89; <0.0001

1 Original data. Within the rows, the means with the same lowercase letter do not differ significantly according to the Student-Newman-Keuls test (*P* = 0.05).

2 Within the columns, the means with the same uppercase letter do not differ significantly by the Student-Newman-Keuls test (*P* = 0.05).

3 Represents the entire time that an aphid spent in the nymphal stage.

**Figure 1:**
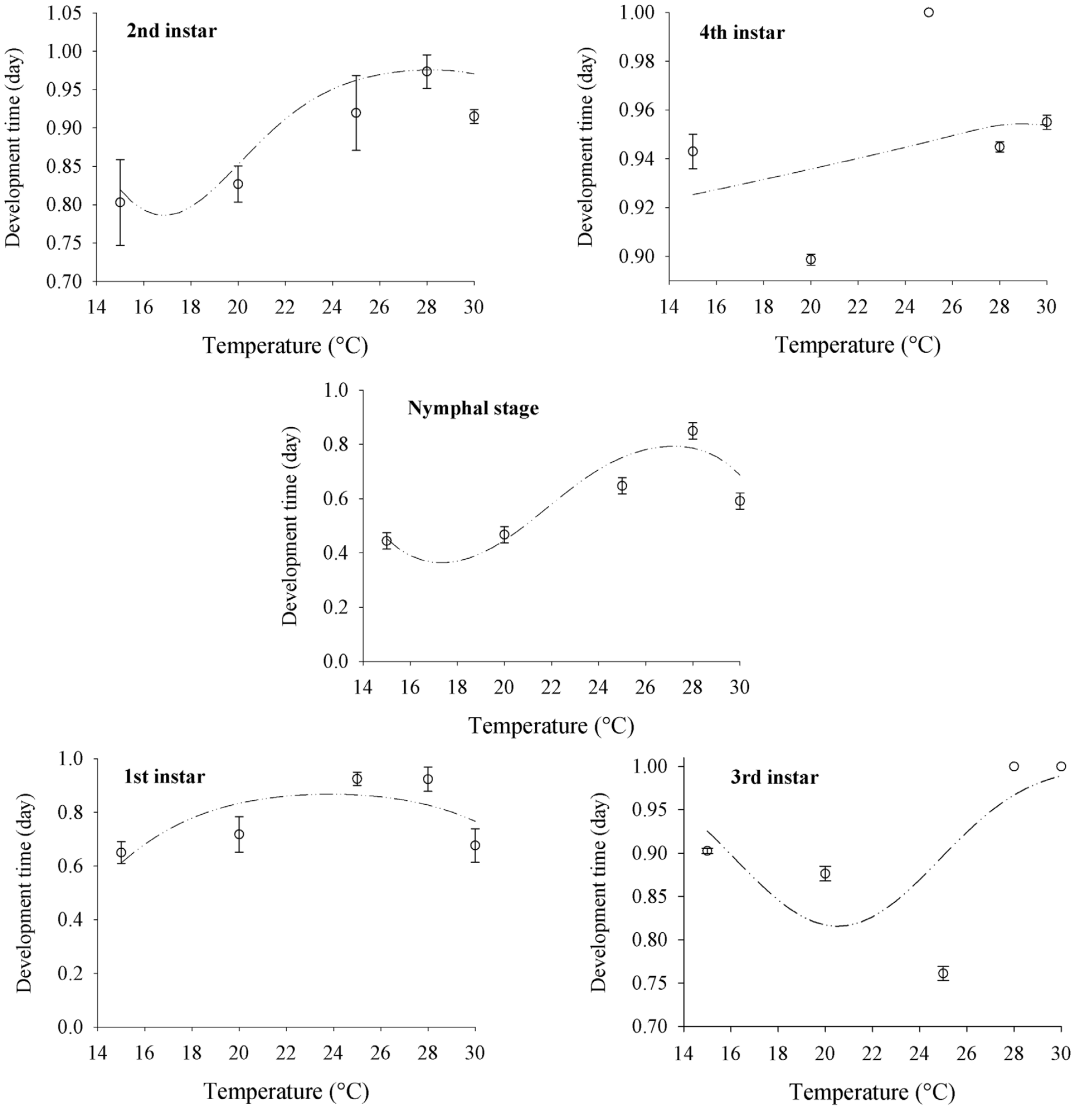
Survival probability of *H. foeniculi* nymphs at different temperatures (15°C, 20°C, 25°C, 28°C or 30°C). The data were subjected to a binomial distribution, and the estimated data were obtained using the model *Pi = 1/(1+exp(−(b_o_+b_1_x+b_2_x^2^…+b_n_x_n_)))*, where *Pi* =  survival probability (survival rate) and *x* =  temperature.

Survival curves for the different instars as a function of temperature were generated based on logistic regression, adjusting the binomial distribution, and the survival probability was estimated using the following model: *Pi = 1/(1+exp(−(b_o_+b_1_x+b_2_x^2^…+b_n_x_n_)))*. The values of *b_o_*, *b_1_* and *b_2_* estimated by logistic regression were as follows: −8.4670 (*b_o_*), 0.8678 (*b_1_*) and −0.0182 (*b_2_*) for the 1st instar; 33.0153 (*b_o_*), −4.7011 (*b_1_*), 0.2229 (*b_2_*) and −0.0033 (*b_3_*) for the 2nd instar; 15.7667 (*b_o_*), −1.3916 (*b_1_*) and 0.00339 (*b_2_*) for the 3rd instar and 2.2766 (*b_o_*), 0.0035 (*b_1_*) and 0.0008 (*b_2_*) for the 4th instar. The resulting survival curves confirmed that the highest survival probabilities for all the instars and the nymph stage of *H. foeniculi* were as follows: 1st instar −0.8673 (at 24°C); 2nd instar −0.9758 (at 27°C); 3rd instar −0.9893 (at 30°C); 4th instar −0.9569 (at 30°C); and nymph stage −0.7934 (at 27°C) (Figure 1).

There was a significant instar-temperature interaction on the instar development time (*F_12, 57_* = 6.43; *P*<0.0001). We observed the influence of temperature on the development times of the 1st (*F_4, 12_* = 56.89; *P*<0.0001), 2nd (*F_4, 12_* = 69.94; *P*<0.0001), 3rd (*F_4, 12_* = 66.76; *P*<0.0001) and 4th (*F_4, 12_* = 163.44; *P*<0.0001) instars, in addition to the development time of the nymph stage (*F_4, 12_* = 215.47; *P*<0.0001). This difference must be temperature dependent because there was a significant difference in nymph development time between the instars only at 15°C (*F_3, 9_* = 43.35; *P*<0.0001), 20°C (*F_3, 9_* = 37.01; *P*<0.0001) and 30°C (*F_3, 9_* = 5.12; *P* = 0.0165) (Table 3).

**Table 3:** Development time (days, mean ± SE) of nymphs in the different instars of *H. foeniculi* at five constant temperatures (15°C, 20°C, 25°C, 28°C or 30°C), a photophase of 12 h and a relative humidity of 70±10%.

Temperature (°C)	Instar^1^^,^ 2				F; Pr	Nymphal stage
	1st	2nd	3rd	4th		(day)^2^^,^ 3
15	2.73±0.16 Ac	5.20±0.29 Ab	4.80±0.25 Ab	6.18±0.22 Aa	= 43.35; <0.0001	18.92±2.41A
20	2.57±0.05 Abc	4.14±0.23 Ba	3.60±0.17 Bb	4.52±0.11 Ba	= 37.01; <0.0001	14.85±1.98B
25	2.39±0.15 Ba	2.28±0.22 Ca	2.27±0.19 Ca	2.14±0.19 Ca	= 0.16; = 0.9235	8.99±1.22C
28	1.53±0.08 Cb	1.83±0.16 Cab	2.05±0.07 Ca	1.94±0.02 Ca	= 5.12; = 0.0165	7.36±1.09D
30	1.09±0.03 Da	1.14±0.08 Da	1.31±0.10 Da	1.25±0.06 Da	= 1.88; = 0.1872	4.80±0.98E
F; Pr	= 56.89; <0.0001	= 69.94; <0.0001	= 66.76; <0.0001	= 163.44; <0.0001	-	= 184.28; <0.0001

1 Within the rows, the means with the same lowercase letter do not differ significantly by the Student-Newman-Keuls test (*P = 0.05*).

2 Within the columns, the means with uppercase letters do not differ significantly by the Student-Newman-Keuls test (*P* = 0.05).

3 Represents the entire time that an aphid spent in the nymphal stage.

The development time of *H. foeniculi* nymphs ranged from 2.73 days (1st instar) to 6.18 days (4th instar) at 15°C, 2.57 days (1st instar) to 4.52 days (4th instar) at 20°C and 1.53 days (1st instar) to 2.05 days (4th instar) at 28°C (Table 3). The development time for nymphs of the 2nd (4.14 days) and 3rd (3.60 days) instars only differed widely at 20°C (Table 3). At other temperatures, the nymph development time did not differ between these two nymph instars (*P* = 0.05) (Table 3). At temperatures between 25 and 30°C, the nymph instar development time ranged from 2.14 days (4th instar) to 2.39 days (1st instar) and from 1.09 days (1st instar) to 1.31 days (3rd instar) (Table 3). However, at these two temperatures, there was no significant difference (*P* = 0.05) in the nymph development times of the four instars of *H. foeniculi* (Table 3). The nymphal period of *H. foeniculi* was, on average, four times longer at the lowest temperature (15°C) compared with the upper limit for survival (30°C).

**Figure 2:**
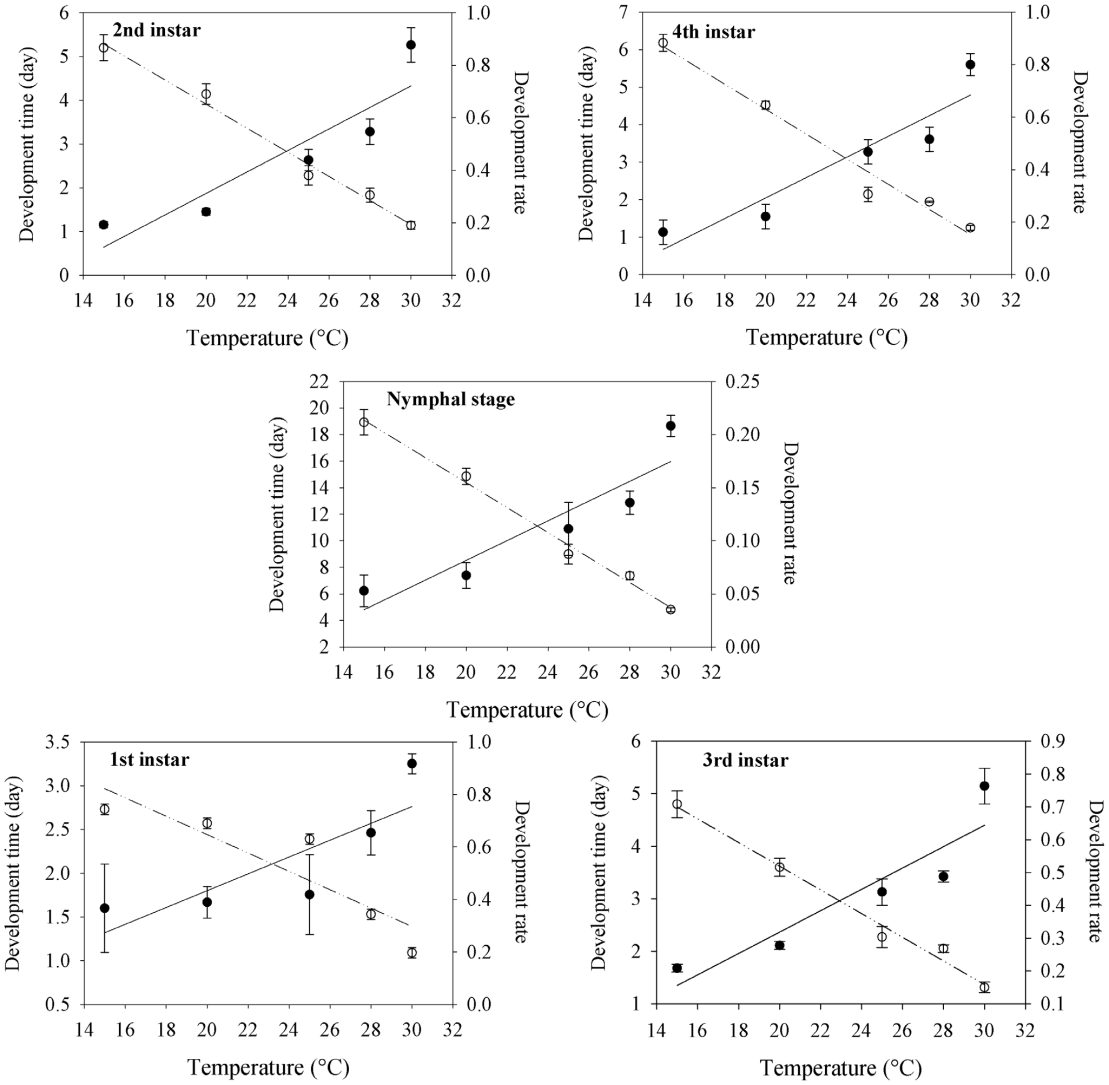
Development time (day) (— — —) and development rate (——) of *H. foeniculi* at five constant temperatures (°C), a photophase of 12 h and a relative humidity of 70±10%. Observed (dots) and estimated (lines) values. Development time (day) and development rate of 1st, 2nd, 3rd and 4th instars, and nymphal stage of *H. foeniculi* as function of temperature (°C).

The developmental rate of *H. foeniculi* nymphs as a function of temperature was adjusted to the linear model obtained by the reciprocal of the hyperbole equation [25] (Table 4 and Figure 2). The estimated values for the base temperature (*T_b_*) and the thermal constant (*k*) for *H. foeniculi* (Table 4 and Figure 2) determined by the hyperbole method [25] were as follows: 6.34°C and 31.25 degree-days (1st instar); 12.60°C and 24.39 degree-days (2nd instar); 10.27°C and 30.30 degree-days (3rd instar); 12.71°C and 25.64 degree-days (4th instar); and 11.24°C and 107.52 degree-days for the nymph stage. The determination coefficient values (*R^2^*) were 0.83 for the 1st instar; 0.70 for the 2nd instar; 0.77 for the 3rd instar; 0.78 for the 4th instar and 0.89 for the nymph stage of *H. foeniculi*. We therefore noted that the models were well adjusted for determining these two parameters (*T_b_* and *k*) (Table 4). The temperature producing the shortest development cycle for the nymph stage of *H. foeniculi* was 30°C (Table 3); however, the highest viability for the nymph stage (85.0%) was observed at 28°C (Table 2).

**Table 4:** Linear regression equations between the rates of development r(T) and temperature (15°C, 20°C, 25°C, 28°C or 30°C) to determine the base temperature (*Tb*) and thermal constant (*K*) for describing the nymph development of *H. foeniculi*.

Instar/Stage	Intercept (a)^1^	Slope (b)^2^	Pr>χ^2^	R^2^	Tb^3^	K^4^
1st	−0.2030	0.0320	<0.0001	0.8330	6.34	31.25
2nd	−0.5170	0.0410	<0.0001	0.7010	12.60	24.39
3rd	−0.3390	0.0330	<0.0001	0.7720	10.27	30.30
4th	−0.4960	0.0390	<0.0001	0.7780	12.71	25.64
Nymph	−0.1046	0.0093	<0.0001	0.8390	11.24	107.52

1 The base temperature or low development threshold (*T_b_*) is calculated as *T_b_* = *−a/b*.

2 The thermal constant, *k* (*day-degrees*) is calculated as *k = 1/b*. *a* is the intercept, and *b* is the regression line slope. The values of *Tb* and *k* were calculated for each instar and nymph stage (from hatching to the adult stage).

## Discussion

The relationship between the insect development rate and temperature was established very early and represents an important ecological variable for modeling the population dynamics of insects [19]. Although insects are not subjected to constant temperatures in nature, controlled laboratory studies can provide valuable insights into the population dynamics of aphids [27].

We confirmed that the survival pattern of *H. foeniculi* is dependent on the temperature and development stage. The survival curves of the different instars as a function of temperature, generated by logistic regression, confirmed that the highest survival rates were found between 24 and 30°C. A higher thermal tolerance was found in 4th instar nymphs, varying between 90 and 100% (Table 2). Although the nymph stage had a shorter duration at 30°C, the viability of the nymph stage was 59%, compromising the aphid's development rate. In contrast, we observed that a constant temperature of 33°C was lethal to *H. foeniculi*. According to Cividanes and Souza [28], the temperature of 30°C was also lethal to nymphs of *Myzus persicae* (Sulzer) (Hemiptera: Aphididae). Nimbalkar et al. [29] reported the survival, albeit at a very low rate (6%), of nymphs of *Aphis gossypii* Glover (Hemiptera: Aphididae) exposed to a temperature of 35°C on cotton plants. According to Campbell et al. [30], higher temperatures lead to greater mortality due to the denaturing of proteins or metabolic disturbances from the accumulation of toxic products; these harmful effects mainly occur if the temperature is held constant. Field studies in northeastern Brazil have found *H. foeniculi* attacking fennel plants during periods with recorded temperatures of over 30°C [31]. This behavior of *H. foeniculi* could be related to the microclimate found inside the flowers and fruits of the host plant, where aphid colonies usually live, or the oscillating day- and night-time thermal conditions to which the nymphs are exposed. The aphid's thermal tolerance can vary according to the species, the aphid biotype and/or the action of endosymbiotic bacteria that, when they undergo single point mutations, can provide amino acids that are rare or non-existent in the phloem sap and confer thermal tolerance to the aphids [32].

The relationships investigated in this study between the biophysical temperature and the development rate and survival are of primary importance for designing integrated *H. foeniculi* control strategies. We observed that a temperature between 15 and 30°C allowed this aphid to develop. However, we did not observe any change in the number of instars. At all the temperatures evaluated, we recorded four instars of *H. foeniculi*, the same number of instars as other insects in the Aphididae family, such as *A. gossypii* [33] and *M. persicae* [28], [34].

The development time patterns for the various stages in juvenile insects can be modified by the temperature changes to which the insect is subjected [35]. For *H. foeniculi*, the effect of temperature on the development time depends on the instar, and instar phases differ in duration at temperatures of 15, 20 and 30°C. Under these conditions, the development times were significantly lower for the 1st instar and significantly higher for the 4th instar. At 20°C, there is a significant difference in the development times of the 2nd and 3rd instar nymphs. Changes in the development times of nymphs of different aphid instars were also observed by Barbosa et al. [34] in *M. persicae* and by Nimbalkar et al. [29] in *A. gossypii*, with the lowest development times for the 4th instar of *A. gossypii* observed at 25°C and for the 2nd and 3rd instars at 30°C.

Non-linear functions can provide more accurate estimates of the relationship between the insect development time and temperature [36], [37]. However, from a practical viewpoint, estimates of the lowest development threshold for an insect by linear extrapolation (Table 4) have proven useful for prediction in numerous studies [38], [39]. These data show that the development times for each instar and nymph stage of *H. foeniculi* vary according to temperature and, consequently, depend on the geographic region and time of year. We can therefore conclude that temperature plays an important role in determining the number of generations of this aphid.

The thermal constants for nymphs of the different instars of *H. foeniculi* were estimated at approximately 25 to 31 degree-days, whereas for the nymph stage, it was estimated to be 108 degree-days. Summing the thermal units (degree-days) required for *H. foeniculi* to complete its development as a function of ambient temperature is useful for describing development rates and predicting the occurrence of population peaks [40].

The base temperatures for the 2nd, 3rd and 4th instars and the nymph stage were estimated at 10 to 13°C. In contrast, the estimated *T_b_* for the 1st instar was 6.34°C, indicating that 1st instar nymphs could be more tolerant than other instars to lower temperatures. For other aphid species, such as *M. persicae* [28] and *Brevicoryne brassicae* (Linné) (Hemiptera: Aphididae) [41], the estimates of *T_b_* were lower than our estimates for *H. foeniculi*. Because aphids with lower thermal thresholds can exploit their plant hosts at lower temperatures [42] and given that the minimum temperatures recorded in northeastern Brazil are higher than the *T_b_* observed for *H. foeniculi* nymphs, our study further confirms that these aphids are potential colonizers of *F. vulgare* in all the phenological phases of the fennel plant in cropping areas in northeastern Brazil.

Based on the development time and viability of *H. foeniculi* nymphs, the temperature of 28°C was considered ideal for nymph development at a constant temperature, accelerating the development time of the nymphal stage (7.0 days) (Table 3) and boosting nymph viability (85.0%) (Table 2).

Accurate determinations of the thermal constant values and the lower and upper developmental thresholds of aphids on a specific crop cultivar would obviously benefit the application of control measures. Because the relationship between the insect developmental rates and temperature is a fundamental component of population dynamics, a realistic description of this relationship is required in physiological models [43] Reliable predictions of *H. foeniculi* population growth would enable the effective application of cultural and chemical controls before the populations reach damaging proportions and migrate to other hosts. Thus, this information is useful for developing phenological models based on relationships involving temperature and development rates, facilitating the prediction of outbreaks of *H. foeniculi* in the fennel crop.
